# Normal and Cystic Fibrosis Human Bronchial Epithelial Cells Infected with *Pseudomonas aeruginosa* Exhibit Distinct Gene Activation Patterns

**DOI:** 10.1371/journal.pone.0140979

**Published:** 2015-10-20

**Authors:** Viviane Balloy, Hugo Varet, Marie-Agnès Dillies, Caroline Proux, Bernd Jagla, Jean-Yves Coppée, Olivier Tabary, Harriet Corvol, Michel Chignard, Loïc Guillot

**Affiliations:** 1 INSERM, UMR_S 938, CDR Saint-Antoine, Paris, France; 2 Sorbonne Universités, UPMC Univ Paris 06, UMR_S 938, CDR Saint-Antoine, Paris, France; 3 Institut Pasteur, Plate-forme Transcriptome Epigenome, Centre Innovation et Recherche Technologiques, Paris, France; 4 Pneumologie pédiatrique, AP-HP, Hôpital Trousseau, Paris, France; The Ohio State University, UNITED STATES

## Abstract

**Background and Aims:**

In cystic fibrosis (CF), *Pseudomonas aeruginosa* is not eradicated from the lower respiratory tract and is associated with epithelial inflammation that eventually causes tissue damage. To identify the molecular determinants of an effective response to *P*. *aeruginosa* infection, we performed a transcriptomic analysis of primary human bronchial epithelial cells from healthy donors (CTRL) 2, 4, and 6 h after induced *P*. *aeruginosa* infection. Compared to noninfected cells, infected cells showed changes in gene activity, which were most marked 6 h postinfection and usually consisted in upregulation.

**Results:**

By comparing for each time point of infection, the transcriptomic response of epithelial cells from CF patients and healthy donors, we identified 851, 638, 667, and 980 differentially expressed genes 0, 2, 4, and 6 h postinfection, respectively. Gene selection followed by bioinformatic analysis showed that most of the differentially expressed genes, either up- or downregulated, were in the protein-binding and catalytic gene-ontology categories. Finally, we established that the protein products of the genes exhibiting the greatest differential upregulation (CSF2, CCL2, TNF, CSF3, MMP1, and MMP10) between CF patients and CTRL were produced in higher amounts by infected cells from CF patients versus CTRL.

**Conclusions:**

The differentially expressed genes in CF patients may constitute a signature for a detrimental inflammatory response and for an inefficient *P*. *aeruginosa* host-cell response.

## Introduction

Cystic fibrosis (CF) is caused by mutations of the gene encoding the cystic fibrosis transmembrane conductance regulator (CFTR), a membrane protein functioning as a chloride channel and expressed at the surface of many epithelia. Defective CFTR function is associated with ion-transport abnormalities that affect the function of a variety of organs. Pulmonary manifestations are usually at the forefront of the clinical picture. CFTR dysfunction in the respiratory epithelia is responsible for dehydration of the airway surface liquid, inspissation of secretions, and deficient mucociliary transport. These abnormalities impair the clearance of inhaled pathogens, thereby allowing opportunistic infections to develop [1]. The bacterial CF microbiota varies significantly with patient age [2, 3]. In young patients, *Staphylococcus aureus* and *Haemophilus influenza* are commonly found. Over time, the opportunistic pathogen *Pseudomonas aeruginosa* gains predominance, contributing over 80% of lung bacteria in adults with CF [4]. This chronic *P*. *aeruginosa* infection and the attendant prolonged inflammatory response cause tissue damage with a progressive decline in lung function that produces most of the morbidity and mortality [5] associated with CF.

The epithelium lining the conducting airways plays a central role in the innate immune response. It is the first barrier against pathogens and possesses several defense mechanisms against colonization by inhaled viruses, fungi, and bacteria [6]. Mechanical factors by which the airway epithelial cells clear pathogens include mucus secretion and the mucociliary escalator. In addition, these cells produce inflammatory mediators involved in the recruitment of cells such as neutrophils to the site of infection, and they are the main source of antimicrobial peptides. Absence of functional CFTR may impair the antimicrobial capabilities of epithelial cells by elevating NaCl concentrations and decreasing the pH of the airway surface liquid [7]. The airways of patients with CF contain high levels of proinflammatory mediators that continuously recruit neutrophils. Although links have been established between epithelial CFTR dysfunction, defective bacteria clearance, and intense inflammatory responses, the relationship between CF airway inflammation and infection remains unclear.

We hypothesized that *P*. *aeruginosa* infection triggers a maladaptive response in the CF epithelial cell mRNA profile. We assessed this hypothesis by comparing the transcriptomic response to *P*. *aeruginosa* infection in CF and normal epithelial airway cells. Our goal was to identify genes that were upregulated and downregulated in the CF cells, compared to the control (CTRL) cells, in response to *P*. *aeruginosa* infection, under the assumption that such changes in gene regulation might explain the strong inflammatory response and chronic *P*. *aeruginosa* infection in patients with CF.

## Materials and Methods

### Human bronchial epithelial cell culture

Human airway epithelial cells from bronchial biopsies (hAECBs) were purchased from Epithelix (Plan-les-Ouates, Switzerland), received at passage 1, and cultured in 75-cm^2^ culture flasks with serum-free Epithelix hAEC culture medium, which was changed every 3 days. One week later, the cells were subcultured in 6-well plates (10^5^ cells/well). After the cells reached confluence, they were incubated overnight in DMEM containing 10% fetal calf serum, 10 mM Hepes, 1% penicillin, and 1% streptomycin before being infected.

### Bacterial strain and growth conditions

The *P*. *aeruginosa* PAK strain used previously [8], expresses the full complement of virulence factors including pili; flagella; the type III secreted exoenzymes S, T, and Y; and a smooth lipopolysaccharide (LPS) belonging to serotype 6.

*P*. *aeruginosa* was grown for 12 h in Luria-Bertani medium, then diluted 4·10^−6^-fold, transferred to fresh medium, and grown overnight to the midlog phase. The culture was centrifuged and the bacterial pellet was washed twice with cold phosphate-buffered saline. The optical density measured at 600 nm was adjusted to give the desired concentration, as previously described [8]. The bacterial count was confirmed by plating serial dilutions on Luria-Bertani agar plates.

### Infection protocol and flowchart of the experimental procedure

We used hAECBs from 4 patients with CF (3 females and 1 male; mean age: 26.5 ± 1.9 years) who were homozygous for the p.F508del mutation and from 4 healthy donors (2 females and 2 males; mean age: 64.5 ± 11.6 years) who had no known diseases or history of smoking (CTRL) (S1 Table). The CF and CTRL hAECBs were simultaneously infected with *P*. *aeruginosa* at 0.25 MOI for 0, 2, 4, 6, and 8 h in DMEM without antibiotics.

At each postinfection time point, supernatants were collected and centrifuged at 3000 × *g* for 15 min to remove the bacteria. Before storage at -80°C, the supernatants were tested for viability of the infected and noninfected cells, by measuring lactate dehydrogenase (LDH) activity (CytoTox 96^®^ NonRadioactive Cytotoxicity Assay, Promega, Madison, WI). Lysis of noninfected cells was used as the basal value to estimate lysis of infected cells. Interleukin (IL)-8 and IL-6 were measured using Duo-Set ELISA kits (R&D Systems, Abingdon, UK) in supernatants to assess epithelial-cell activation by *P*. *aeruginosa*.

Cell lysates were stored at -80°C until RNA isolation (see below). The transcriptomes of the 4 CF and 4 CTRL specimens were analyzed 0, 2, 4, and 6 h postinfection (32 analyses in all). Protein expression was assessed 8 h postinfection. We first evaluated gene regulation in CTRL cells infected with *P*. *aeruginosa* versus nonstimulated cells. Then, we compared the mRNA expression profiles of infected CF versus CTRL cells at each postinfection time point.

### RNA and protein isolation

Small RNAs (< 200 nt), large RNAs (> 200 nt), and proteins were isolated in three separate fractions, using the NucleoSpin^®^ miRNA kit (Macherey-Nagel, Düren, Germany).

### RNA purification and library construction

We used 2 to 5 μg of large RNA to purify polyadenylated mRNAs and to build an RNA library, using TruSeq RNA Sample Prep Kit v2 (Illumina, #RS-122-2001 and #RS-122-2002, San Diego, CA) as recommended by the manufacturer. The nondirectional libraries thus obtained were CTRL led by Bioanalyzer DNA1000 Chips (Agilent Technologies, #5067–1504, Santa Clara, CA) and quantified using spectrofluorimetry (Quant-iT™ DNA High-Sensitivity Assay Kit, #Q33120, Invitrogen, Life Technologies, Carlsbad, CA).

### Sequencing and Bioinformatic analysis

Sequencing of the 32 samples was performed on the HiSeq 2000 sequencer (Illumina) in single-end mode in order to have approximately 100 million reads of 50 bases per sample.

Reads were cleaned of adapter sequences and low-quality sequences using an in-house program (https://github.com/baj12/clean_ngs). Only sequences at least 25 nt in length were considered for further analysis. TopHat (version 1.4.1.1, default parameters, http://ccb.jhu.edu/software/tophat) was used for alignment on the reference genome (hg19). Genes were counted using HTseq-count (parameters: -m intersection-nonempty, -s yes, -t exon) [9, 10].

The PANTHER (Protein ANalysis THrough Evolutionary Relationships) (http://www.pantherdb.org/) Classification System was used to classify genes identified in our transcriptomics data according to their molecular function annotated using ontology terms [11, 12].

### Multiplex detection immunoassays

To confirm that genes of interest showed significant differences in expression between CF and CTRL cells, protein levels were measured in stimulation supernatants, using the Human Magnetic Luminex Screening Assay (R&D Systems). We used an 11-plex panel to assay cytokines, chemokines, and growth factors; and a 3-plex panel to assay matrix metalloproteinase (MMP) levels.

### Statistical analysis

#### Cytokines

To determine whether differences between the CF and CTRL groups were statistically significant, we used Prism 6.00 software (GraphPad Software, La Jolla, CA), as indicated in the figure legends. *P* values <0.05 were considered significant. ANOVA was performed to compare quantitative variables across groups. To correct for multiple testing, we used either Bonferroni’s method (comparisons of all groups) or Dunnett’s method (comparisons of groups vs. a CTRL condition).

#### RNAseq data

Counts were analyzed using R version 3.0.2 and the Bioconductor package DESeq2 version 1.2.9 (www.bioconductor.org) [13]. Data were normalized using DESeq2 and the default parameter. To estimate dispersion and test for differential expression, we used the default parameters (including outlier detection and independent filtering). The generalized linear model was set with time, condition (CF vs. CTRL), and patient as the main effects and with time x condition and condition x patient as the interaction terms, since the patient effect was nested within the condition effect and cells from each patient were studied at four different time points. To avoid model overfitting, patients were designated by the same label in each condition, and their nesting within the condition was included in the model through the condition x patient interaction term. Extracted contrasts included comparisons between the CF and CTRL groups at each time point and pairwise comparisons of time points in each condition. Raw *P* values were adjusted for multiple testing according to the Benjamini and Hochberg procedure, and genes with an adjusted *P* value <0.001 were considered differentially expressed [14].

## Results

### Activation of primary bronchial epithelial cells (hAEBCs) by *P*. *aeruginosa*

**Fig 1:**
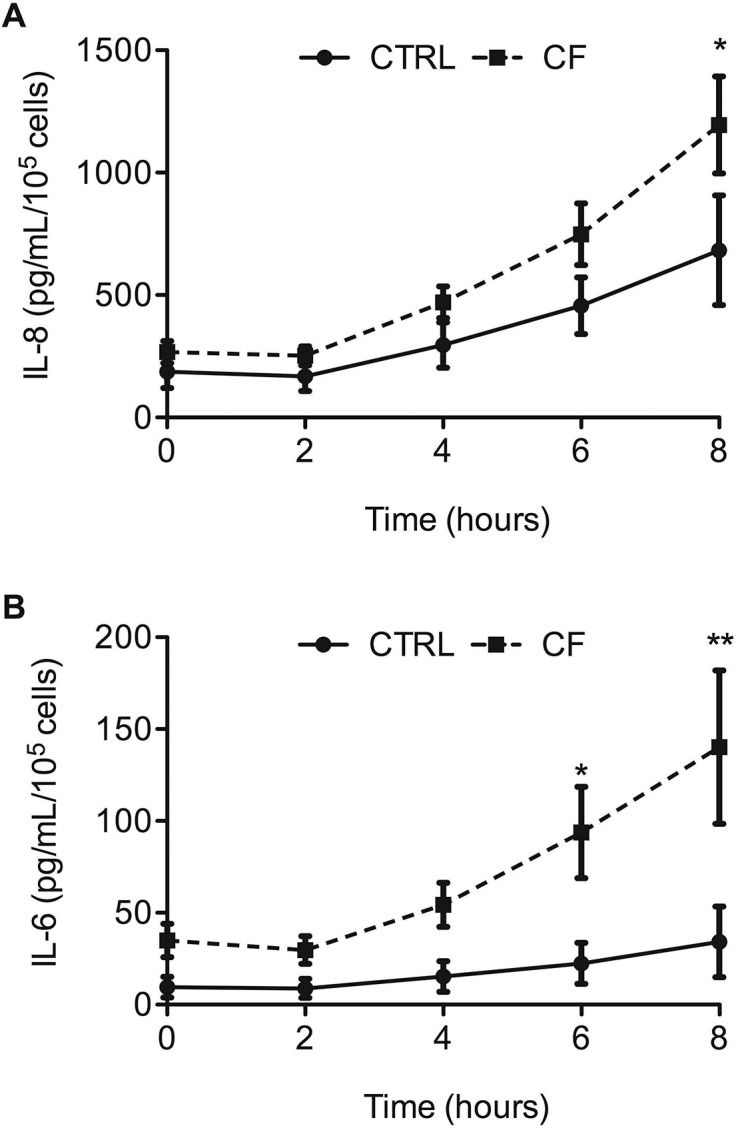
Validation of activation of primary bronchial epithelial cells (hAEBCs) upon *P*. *aeruginosa* infection. Kinetics of IL-8 (A) and IL-6 (B) production by hAEBCs infected with *P*. *aeruginosa*. At each time point, supernatants were collected for ELISAs of IL-8 and IL-6. The data are mean±SEM of IL-8 or IL-6 concentrations for four different patients. The statistical analysis consisted in ANOVA followed by Bonferroni’s multiple comparison test. *P<0.05 and **P<0.001. Each value in the cystic-fibrosis (CF) group was compared with the value at the same time point in the CTRL group.

After infection, the kinetics of IL-8 and IL-6 production were comparable for CF and CTRL cells (Fig 1A and 1B, respectively). However, IL-6 and IL-8 concentrations differed significantly between CF and CTRL cells from 6 hours and at 8 hours postinfection, respectively. These data were obtained under experimental conditions in which *P*. *aeruginosa* infection did not alter the viability of CF or CTRL cells, as assessed based on LDH release (data not shown).

### Transcriptional response of CTRL primary bronchial epithelial cells (hAEBCs) to *P*. *aeruginosa*

When we analyzed the transcriptomic response of CTRL cells to *P*. *aeruginosa* infection, we found a significantly greater number of upregulated than of downregulated genes, compared to noninfected CTRL cells: 2, 4, and 6 h postinfection, the numbers of upregulated genes were 0, 13, and 186; whereas the numbers of downregulated genes were 0, 11, and 25; respectively (*P*<0.001).

Among the differentially expressed genes, we selected those whose fold change (FC) was ≥2 in the event of upregulation and ≤0.5 in the event of downregulation: 75 upregulated genes and 6 downregulated genes (S2 Table) were thus selected.

PANTHER (Protein ANalysis THrough Evolutionary Relationships) was used to classify genes according to gene-ontology molecular functions. Upregulated genes fell into four main functional categories: protein binding (37.1% of upregulated genes), catalytic activity (13.5%), nucleic-acid-binding transcription-factor activity (12.4%), and receptor activity (9%). The number of downregulated genes was too small for a meaningful classification.

Among upregulated genes in the protein-binding category, those having the greatest fold change values encoded the transcription factors Kruppel-like factor 2 (*KLF2*) and Nuclear receptor subfamily 1, group D, member 1 (*NR1D1*); the cytokine IL-17C; the chemokine (C-C motif) ligand 20 (*CCL20*); the hormonally active protein adrenomedullin 2 (*ADM2*); and the apoptosis inhibitor baculoviral IAP repeat-containing protein 3 (*BIRC3*) (Table 1).

**Table 1:** List of genes in the protein-binding gene-ontology category that were most upregulated (FC ≥ 3) in CTRL cells 6 hours after *P*. *aeruginosa* infection.

Gene	GO molecular function	CTRL 0 h	CTRL 6 h	FC	adjpBH
*KLF2*	sequence-specific DNA binding transcription factor activity	62	583	8.3	1.3^E-17^
*IL17C*	cytokine receptor binding	8	75	5.3	3.1^E-06^
*CCL20*	chemokine activity	583	2094	3.7	1.6^E-08^
*ADM2*	hormone activity	372	1278	3.4	1.1^E-04^
*NR1D1*	ligand-activated sequence-specific DNA binding RNA polymerase II transcription factor activity	940	3232	3.3	4.0^E-12^
*BIRC3*	peptidase activity ;protein binding ;peptidase inhibitor activity	598	1862	3	4.5^E-08^

Genes were classified according their molecular function, using the PANTHER Classification System (http://www.pantherdb.org/). 0 h, nonstimulated; 6 h, 6 hours postinfection; FC, fold change; adjpBH, adjusted *P* value to which the α cutoff was applied.

Among the downregulated genes, we identified three genes having known molecular functions, namely, the two transcriptional regulators early growth response protein 1 (*EGR1*) and SMAD family member 6 (*SMAD6*); and the nucleosome assembly protein (NAP) family member NAP 1-like 3 (*NAP1L3*).

### Differential gene activation in cystic fibrosis (CF) versus CTRL cells in response to *P*. *aeruginosa* infection

**Fig 2:**
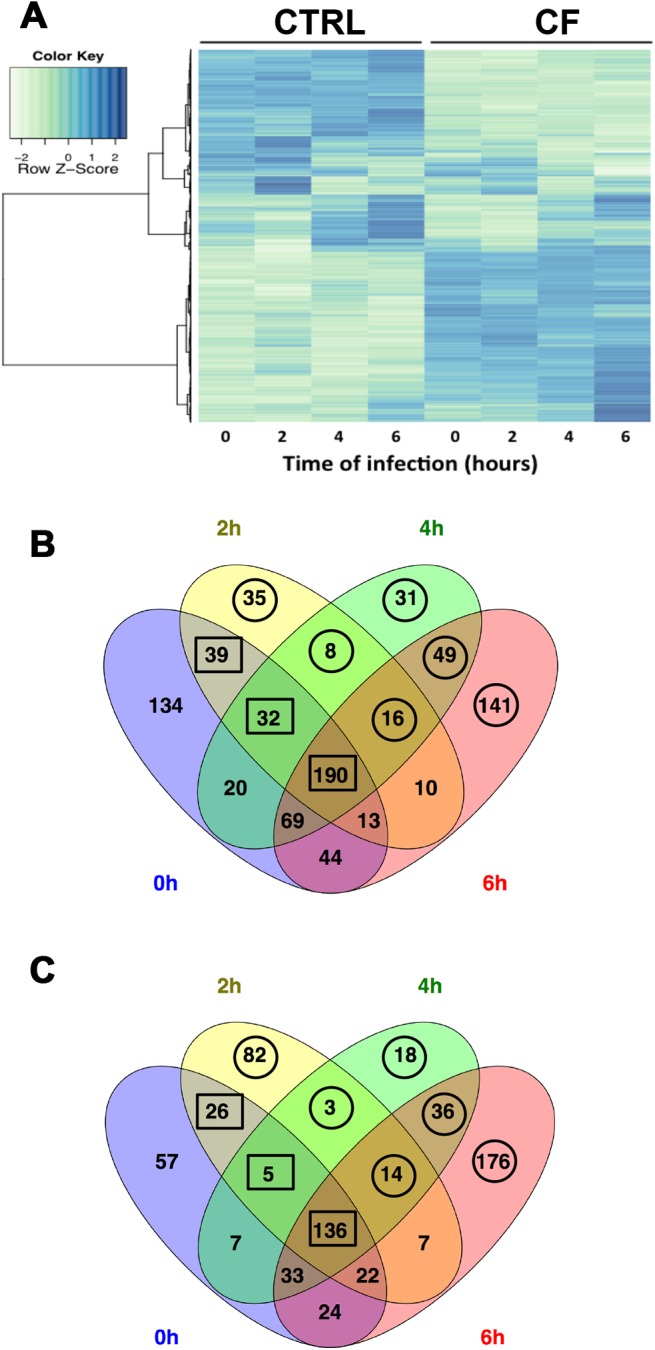
Overview of gene expression profiles. A- The heat-map of the mean expression levels of all the genes in each condition revealed two distinct clusters that separated cystic-fibrosis (CF) cells from CTRL cells. B and C- Venn diagram of differentially expressed genes between cystic-fibrosis and CTRL cells upon *P*. *aeruginosa* infection. Circles: Differentially expressed genes upregulated (B) or downregulated (C) at a single postinfection time point or at two or three consecutive postinfection time points (thus, 0h was not considered). We selected the genes whose fold-change in expression level was ≥2 in the event of upregulation and ≤0.5 in the event of downregulation. Squares: Differentially expressed genes upregulated (B) or downregulated (C) at two, three, or four consecutive time points (thus, 0h was considered). We selected the genes for which the ratio of FC in infected cells over FC in noninfected cells was ≥1.5 in the event of upregulation or ≤0.6 in the event of downregulation.

We compared genes that were differentially expressed in CF and CTRL cells at each postinfection time point. The heat-map of the mean gene-expression level under each condition revealed two distinct clusters that separated CF and CTRL cells, one of upregulated genes and the other of downregulated genes (Fig 2A).

We then identified the genes whose level of expression differed between CF and CTRL cells with *P*<0.001. The numbers of these genes were 851, 638, 667, and 980 at the four postinfection time points (0, 2, 4 and 6 h), respectively (data not shown).

An MA plot (transcripts data have been transformed onto the M (log ratios) and A (mean average) scale) was created to visualize differences in gene expressions between CF and CTRL cells at each postinfection time point. Among the differentially expressed genes 541, 343, 415, 532 were upregulated and 310, 295, 252, and 448 were downregulated at 0, 2, 4, and 6 h postinfection, respectively (data not shown).

We then created Venn diagrams representing upregulated (Fig 2B) and downregulated genes (Fig 2C). We identified genes that were upregulated or downregulated (134 and 57, respectively) only at baseline (0h). These genes were not considered further, since our goal was to study gene expression changes induced by *P*. *aeruginosa* infection. We then separated the genes differentially expressed at a single postinfection time point and those differentially expressed at two or three consecutive time points (i.e., 0, 2, 4, 6; or 0, 2, 4; or 0, 2; or 2, 4, 6; or 4, 6; or 2, 4 h). To exclude genes whose differences in expression between CF and CTRL cells were similar at baseline (0 h) and after infection, and therefore probably related to the *CFTR* genotype and not to *P*. *aeruginosa* infection, for each gene, we computed the ratio of the fold change value at each postinfection time point over the fold change value at 0h. We then selected the genes for which this ratio was ≥1.5 (arbitrary choice) in the event of upregulation and ≤0.6 (arbitrary choice) in the event of downregulation, at one or more postinfection time points. For genes differentially expressed at a single time point or at two or three consecutive time points (2 and 4 h; or 2, 4 and 6 h; or 4 and 6 h), we defined a significant change in gene expression as fold change ≥2 in the event of upregulation and fold change ≤0.5 in the event of downregulation. These criteria identified 171 upregulated genes and 236 downregulated genes in CF versus CTRL cells, which are listed in S2 Table.

### Genes differentially upregulated in cystic fibrosis (CF) versus CTRL epithelial cells infected with *P*. *aeruginosa*

PANTHER was then used to identify key functions differentially affected by the response to infection of CF and CTRL cells. In all, seven gene-ontology molecular-function categories were overexpressed in CF compared to CTRL cells after *P*. *aeruginosa* infection (Table 2).

**Table 2:** Gene-ontology molecular-function categories upregulated in infected cystic-fibrosis cells compared to CTRL cells.

GO molecular function	Category name	Genes (n)	% gene hit against total genes
0005488	Binding	53	34.6
0003824	Catalytic activity	39	25.5
0004872	Receptor activity	16	10.5
0030234	Enzyme regulator activity	15	9.8
0005198	Structural molecule activity	8	5.2
0005215	Transporter activity	7	4.6
0001071	Nucleic acid binding transcription factor activity	5	3.3

The two main categories were protein binding and catalytic activity, with 34.6% and 25.5% gene hits vs. total genes, respectively. The five genes the most differentially expressed in the protein-binding and catalytic-activity categories are reported in Table 3A and 3B, respectively.

**Table 3:** Genes in the protein-binding (A) and in the catalytic-activity (B) gene-ontology category: five upregulated with the greatest difference in expression level between cystic-fibrosis and CTRL cells after infection.

**A**									
**Gene**	**GO molecular function**	**FC 0 h**	adjpBH	**FC 2 h**	adjpBH	**FC 4 h**	adjpBH	**FC 6 h**	adjpBH
*IL-17C*	cytokine receptor binding					**6.1**	2.3^E^-05	**5.8**	7.3^E^-06
*CSF3*	cytokine activity	**6**	2.3^E-13^	**4.6**	6.3^E^-07	**5.9**	9.9^E^-10	**11.4**	2.9^E^-18
*SIRPB1*	chemokine activity	**13.4**	3.5^E-31^	**23.6**	1.2^E^-28	**13.6**	9.4^E^-25	**9.8**	1.5^E^-16
*TNF*	TNF receptor binding cytokine activity	**2.4**	6.1^E-05^	**3.2**	2.0^E^-06	**3.5**	9.3^E^-08	**5**	3.9^E^-13
*CCL2*	chemokine activity	**2.6**	1.8^E-09^	**2.1**	1.7^E^-04	**2.9**	1.4^E^-10	**5**	2.4^E^-24
**B**									
**Gene**	**GO molecular function**	**FC 0 h**	adjpBH	**FC 2 h**	adjpBH	**FC 4 h**	adjpBH	**FC 6 h**	adjpBH
*MMP1*	metallopeptidase activity	**3.1**	7.7^E-06^	**3.1**	3.3^E^-04	**5.1**	5.2^E^-09	**5.8**	1.1^E^-10
*SERPINA3*	serine-type peptidase, peptidase inhibitor activity							**5.3**	9.6^E^-06
*MMP13*	metallopeptidase activity	**2.2**	6.3^E-06^	**2.8**	1.1^E^-07	**3.8**	9.1^E^-15	**4.6**	1.0^E^-19
*PDGFB*	protein kinase activity, receptor binding	**2.5**	2.3^E-07^	**3.5**	4.1^E^-11	**3.7**	1.8^E^-12	**4.5**	2.1^E^-16
*MMP10*	metallopeptidase activity					**2.8**	8.4^E^-07	**3.5**	3.4^E^-10

FC, fold change; h, hours; adjpBH, adjusted *P* value to which the α cutoff was applied.

We then focused on genes for which theoretical considerations suggested a role in the intense inflammatory response to infection seen in patients with CF. Some of these genes belonging to protein-binding family were involved in the inflammatory response, such as tumor necrosis factor (TNF), IL-17C, colony-stimulating factor (CSF) 2 (also called GM-CSF), CSF3 (also called G-CSF), and CCL2. Others code for proteolytic enzymes classified in the catalytic category, namely MMP1, MMP10, and MMP13.

IL-17C and CSF2 mRNA expressions were differentially upregulated in CF cells 4 and 6 h postinfection, respectively. TNF, CSF3, and CCL2 mRNAs were already differentially upregulated in CF cells at baseline (0 h) and the difference was increased 4 h postinfection. Similarly, MMP1 and MMP13 mRNAs showed significant differential upregulation in CF cells at 0h, with increases in the differences 4 h postinfection. By contrast, MMP10 was differentially upregulated only at the 4 h postinfection time point.

**Fig 3:**
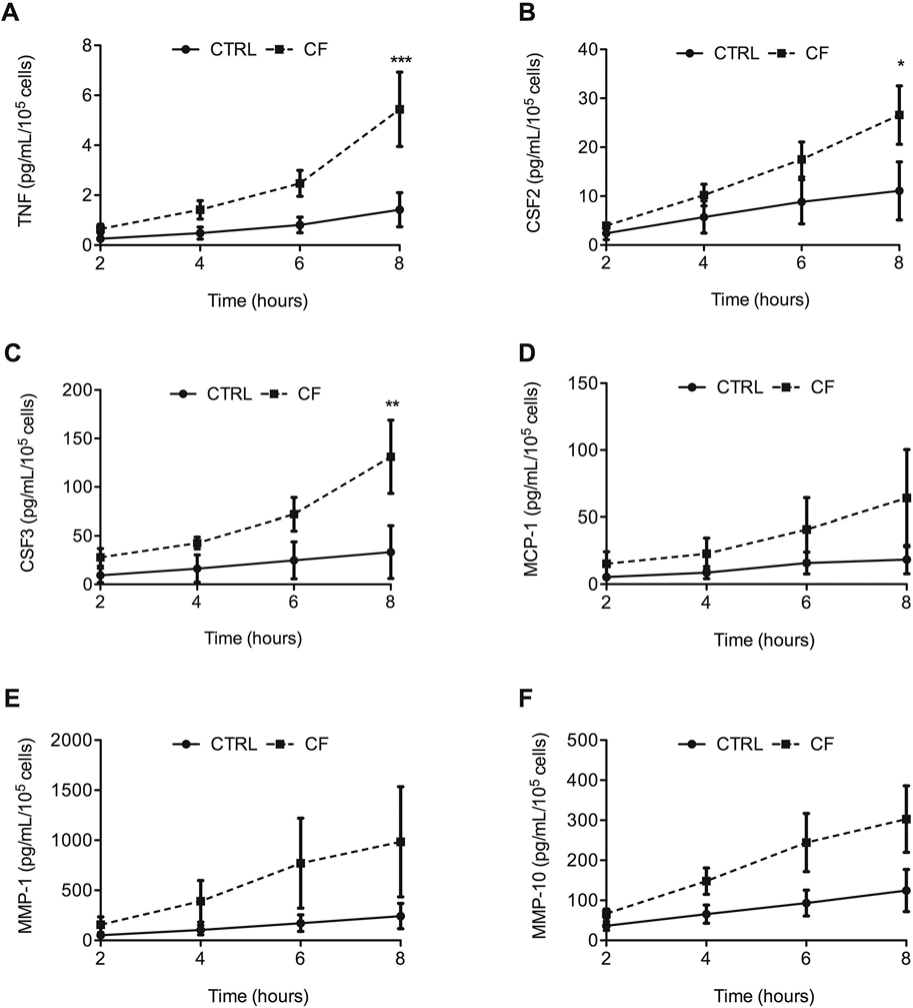
TNF (A), CSF3 (B), CSF2 (C), CCL2 (D), MMP1 (E), and MMP10 (F) protein synthesis in response to *P*. *aeruginosa* infection of cystic-fibrosis (CF) and CTRL cells. Protein levels were measured in supernatants of infected CF and CTRL cells 0, 2, 4, 6, and 8 hours postinfection, using the Human Magnetic Luminex Screening Assay. Data are mean±SEM of cytokine concentrations for four different patients. The statistical analysis consisted in ANOVA followed by Bonferroni’s multiple comparison test. *P<0.05 and **P<0.001. Each value in the cystic-fibrosis (CF) group was compared with the value at the same time point in the CTRL group.

Among proteins (Fig 3), IL-17C and MMP13 were not detected, even 8 h postinfection. TNF and CSF3 protein levels differed significantly between CF and CTRL cells 8 h postinfection. After infection, particularly at the 6 h and 8 h time points, CSF2, CCL2, MMP1, and MMP10 proteins were expressed at higher levels in CF than CTRL cells, although the differences were not statistically significant.

We detected significant differential production of IL-8 and IL-6 proteins between CF and CTRL cells (see Fig 1). As expected, the transcriptional analysis shows significant induction of IL-8 or IL-6 mRNA expression (*P*<0.05).

To explain the excessive inflammation triggered by *P*. *aeruginosa* in patients with CF, we assessed the mRNA expression level of Pattern Recognition Receptors (PRRs) known to be involved in *P*. *aeruginosa* recognition. *P*. *aeruginosa* expressed numerous pathogen-associated molecular patterns (PAMPs) such as LPS recognized by toll-like receptors (TLRs) 2, 4 and flagellin recognized by TLR5. None of the PPRs met the stringent *P* or fold change criteria used in our study. Nevertheless, some PRRs may be involved in the upregulation of proinflammatory genes. Thus the mRNAs for TLR2, 4, and 6 were significantly (*P*<0.05) upregulated in CF cells both at 0 h and after *P*. *aeruginosa* infection (S1 Fig). Other PRRs showed nonsignificant differences in expression: although TLR9 was upregulated and TLR5 downregulated, in CF cells compared to CTRL cells. Nucleotide-binding oligomerization domain containing 2 (NOD2) was similarly induced by infection in CF and CTRL cells

### Genes differentially downregulated in cystic fibrosis (CF) versus CTRL epithelial cells infected with *P*. *aeruginosa*

PANTHER identified a total of 10 gene-ontology molecular-function categories that were expressed at lower levels in CF cells than in CTRL cells (Table 4).

**Table 4:** Gene-ontology molecular-function categories downregulated in infected cystic-fibrosis cells compared to CTRL cells.

GO molecular function	Category name	Genes (n)	% gene hit against total genes
0003824	Catalytic activity	60	33
0005488	Binding	50	27.5
0005215	Transporter activity	16	8.8
0001071	Nucleic acid binding transcription factor activity	14	7.7
0004872	Receptor activity	13	7.1
0005198	Structural molecule activity	13	7.1
0030234	Enzyme regulator activity	11	6
0045182	translation regulator activity	3	1.6
0016209	Antioxidant activity	1	0.5
0000988	Protein binding transcription factor activity	1	0.5

Catalytic activity and protein binding were the two main underexpressed categories, with 33% and 27.5% of genes, respectively. The five genes in the catalytic-activity and protein-binding categories exhibiting the largest differences in expression between CF and CTRL cells are listed in Table 5A and 5B, respectively.

**Table 5:** Genes in the catalytic-activity (A) and in the protein-binding (B) gene-ontology category: five downregulated genes with the greatest difference in expression level between cystic-fibrosis and CTRL cells after infection.

**A**									
**Gene**	**GO molecular function**	**FC 0h**	adjpBH	**FC 2h**	adjpBH	**FC 4h**	adjpBH	**FC 6h**	adjpBH
USP9Y	cysteine-type peptidase activity, ubiquitin-protein, ligase activity, mRNA binding	**0.13**	9.2E-09	**0.12**	1.1E-04	**0.1**	3.5E-06	**0.08**	9.7E-08
ATP12A	hydrolase activity, ion channel activity, cation transmembrane transporter	**0.23**	1.8E-13	**0.32**	1.5E-06	**0.25**	5.3E-10	**0.12**	4.2E-24
KPRP	ligase activity, structural constituent of cytoskeleton	**0.33**	6.4E-07	**0.17**	2.0E-11	**0.22**	7.7E-11	**0.14**	1.1E-15
KLK12	serine-type peptidase activity	**0.16**	3.7E-10	**0.07**	2.1E-12	**0.16**	4.6E-07	**0.17**	1.3E-06
KLK13	serine-type peptidase activity	**0.3**	1.1E-22	**0.24**	5.2E-25	**0.24**	2.8E-30	**0.18**	8.6E-42
**B**									
**Gene symbol**	**GO molecular function**	**FC 0h**	adjpBH	**FC 2h**	adjpBH	**FC 4h**	adjpBH	**FC 6h**	adjpBH
ADM2	hormone activity	**0.21**	2.0E-08	**0.27**	4.7E-04	**0.09**	2.0E-14	**0.08**	3.5E-16
EIF1AY	translation initiation factor activity	**0.09**	9.6E-10	**0.05**	3.3E-06	**0.06**	1.8E-06	**0.09**	1.3E-05
NLGN4Y	protein binding	**0.13**	2.6E-07	**0.09**	4.5E-05	**0.09**	2.0E-05	**0.1**	2.6E-05
ELF5	sequence-specific DNA binding, transcription factor activity receptor binding	**0.22**	2.3E-12	**0.15**	1.8E-10	**0.2**	2.6E-11	**0.11**	1.6E-20
TNNC1	structural constituent of cytoskeleton, calcium ion and calmodulin binding			**0.12**	1.9E-04				

FC, fold change; h, hours; adjpBH, adjusted *P* value to which the α cutoff was applied.

Genes in the catalytic-activity category included kallikreins (KLK12 and KLK13) and ubiquitin-specific peptidase 9, Y-linked (USP9Y), which are enzymes exhibiting peptidase activity. Genes in the protein-binding category had a variety of functions, such as hormonal (ADM2), transcriptional (E74-like factor 5 [ELF5]), translational (eukaryotic translation initiation factor 1A, Y-linked [EIF1AY]), and calcium-ion binding (troponin C type 1 [TNNC1]) activities. For most of these genes, the difference in downregulation between CF and CTRL cells was significantly larger 6 h postinfection than at other time points; the exceptions were KLK12 and EIF1AY, for which the differences were largest 2 h postinfection.

## Discussion

The respiratory tract epithelium is a critical environmental interface that participates to the regulation of the innate immune response of the host to infection. Host-pathogen interactions determine the intensity of the innate inflammatory response and, therefore, the risk of tissue injury. Our primary objective was to characterize the transcriptomic response of healthy primary bronchial epithelial cells to *P*. *aeruginosa* infection, in order to identify effectors of the innate immune response involved in combatting bacterial infections. We also planned to compare this transcriptomic response with that of infected epithelial cells from patients with CF. We reasoned that differences between normal and CF cells might point to abnormalities in innate immune function responsible for failure of CF epithelial cells to eradicate *P*. *aeruginosa*, as well as for the generation of an exaggerated inflammatory response.

We first investigated primary bronchial epithelial cells from healthy CTRL to assess their transcriptional response to alive *P*. *aeruginosa* infection. We performed tests infection of CTRL cells at 2, 4, 6 h to allow sufficient time for expression of *P*. *aeruginosa* virulence factors and of host genes and then compared gene expressions of infected to non-infected cells (0 h). The response was strongest 6 h postinfection; in contrast, after 2 h the transcriptome of *P*. *aeruginosa*-infected epithelial cells was unchanged. After 4 h, only two upregulated and three downregulated genes showed an at least 2-fold increase or decrease in their expression level versus uninfected cells. After 6 h, the same criterion was met by 75 upregulated and 6 downregulated genes.

The upregulated genes included genes encoding innate immunity effectors, such as IL-17C, CCL20, and TNF, which contribute to combat bacterial infections. The gene for the transcription factor KLF2 was also upregulated. A previous study established that KLF2 overexpression was induced by ExoS, a toxin delivered through the *P*. *aeruginosa* type III secretion system and associated with epithelial-cell death [15].

Among downregulated genes, we found *NAP1L3*, *SMAD6*, cell division cycle-associated protein 7 (*CDCA7*), *EGR1*, and zinc finger protein 367 (*ZNF367*). SMAD6 promotes the antiinflammatory response by playing an important role in transforming growth factor-beta 1 (TGF-ß1)-mediated negative regulation of proinflammatory signaling [16]. *SMAD6* downregulation during Gram-negative bacterial infection has been reported previously: the bacterial endotoxin LPS can inhibit the TGF-ß1 signaling pathway leading to *SMAD6* expression [17]. EGR1 is a nuclear protein that functions as a transcriptional regulator. The EGR1 downregulation observed in our infected CF cells may be ascribable to acyl-homoserine lactones, which are intercellular signaling molecules used in quorum sensing by *P*. *aeruginosa* and *Burkholderia cepacia* [18].

Previous studies have identified genes whose expression in respiratory epithelial cells changed after *P*. *aeruginosa* infection. High-density DNA microarrays have been used to identify regulated genes in A549 cells infected with live PAK *P*. *aeruginosa* then studied for 3 h [19]. An at least 2-fold change in the expression level was found for only 22 upregulated genes and 2 downregulated genes. Some of these genes were also upregulated in our study such as Ras homolog family member B (*RHOB*), E74-like factor 3 (*ELF-3* or *ESE-1*), nuclear factor of kappa light polypeptide gene enhancer in B-cells inhibitor, alpha (*NFKBIA* or *MAD3*), and macrophage cationic peptide 1 (*MCP-1*)). In contrast, the two downregulated genes were not found. Serial analysis of gene expression (SAGE) has been used for a transcriptomic analysis of primary bronchial epithelial cells infected with heat-inactivated *P*. *aeruginosa* (PAO1 strain) for 6 h [20]. The results indicated regulation of four categories of genes: keratins, proteinase inhibitors, IL-1 family members, and S100 calcium-binding proteins. None of these genes were regulated in our study. These discrepancies across studies may be ascribable to differences in respiratory epithelial cell types (e.g., bronchial, alveolar, immortalized, or primary cells), bacteria (strains, inactivated vs. alive), MOI, time of infection, or even expression-profiling techniques.

The transcriptomic response of CTRL cells to *P*. *aeruginosa* may constitute the signature of the ideal immune response of respiratory epithelial cells to infection. To identify gene expression differences that might explain the inadequate innate immune response of CF cells to infection, we compared the transcriptomes of CF and CTRL primary bronchial epithelial cells infected with *P*. *aeruginosa*. The two groups of cells were exposed to identical experimental conditions, to avoid introducing confounding factors. An important difference with our data concerning the response of infected CTRL cells, for which a small number of gene expressions were modulated, is the large number of genes whose expression levels differed between CF and CTRL cells. After *P*. *aeruginosa* infection, many genes whose expression was not altered in CTRL cells showed upregulation or downregulation in CF cells.

Interestingly, the proinflammatory genes that were upregulated in CF cells compared to CTRL cells included the genes encoding IL-17C, TNF, CSF2, CSF3, and CCL2, all of which are major mediators of inflammation. However, protein expression was not evidenced for all of these genes. Thus, IL-17C was not detected in supernatants of CF or CTRL cells, even 8 h postinfection. After 6 h, among mRNA levels, those for TNF were higher than those for CCL2 and CSF2, and similar to those for CSF3. These differences were not reflected in the protein concentrations in the supernatants: TNF protein concentrations were very low (7 and 13 pg/mL after 6 h and 12 and 28 pg/mL after 8 h in CTRL and CF cell supernatants, respectively); whereas CSF2, CCL2, and CSF3 protein levels were high. Nonetheless, 8 h postinfection, TNF and CSF3 protein concentrations were significantly higher in CF than in CTRL cells, in keeping with the results of the transcriptomic analysis. CSF2 and CCL2 protein levels were higher in CF cells than in CTRL cells and increased further with time after infection, although the differences were not statistically significant. In general, for the genes with the greatest differences in expression, we also found differences in protein levels. CSF2 and CSF3 are involved in the host response to microbial infections [21, 22]. CSF2 secretion promotes neutrophil maturation and activation [23]. Thus, CSF2 activates the Jak/STAT pathway, thereby delaying spontaneous neutrophil apoptosis [24]. CSF3 may contribute to lung inflammation, as it increases neutrophil chemotactic activity. Whereas the production of CSF2 is well documented, less is known about the regulation of CSF3 in airway epithelial cells. In one study, lung epithelial cells stimulated by LPS from *P*. *aeruginosa* released CSF3 [25]. These findings are consistent with the presence in CF of airway inflammation characterized by a profuse influx of neutrophils into the lungs.

The main family of mediators in the catalytic category is the MMP family. MMPs are zinc-dependent enzymes with proteolytic activity against a wide range of extracellular proteins. MMPs are therefore involved in extracellular matrix turnover, tissue degradation, and tissue repair [26]. MMPs are anchored to the cell surface or secreted as proenzymes that are activated by proteolytic cleavage [27]. Their potential substrates include membrane proteins and proteins in the extracellular compartment. MMP activity is controlled by specific tissue inhibitors of metalloproteases (TIMPs) [28]. MMPs are expressed in a variety of normal and disease processes, such as development, involution, repair, inflammation, and tumor growth. They play a major role in regulating the host immune response to infections. By degrading extracellular matrix components and by modulating cytokine and chemokine activity, MMPs allow the migration of inflammatory cells from the bloodstream and drive their recruitment [29–31]. In our model, MMP1, MMP10, and MMP13 mRNAs, which were upregulated, showed the greatest differences between CF and CTRL cells. MMP1 and MMP10 proteins were detected in the supernatants of infected cells; in contrast, MMP13, although described as a secreted MMP, was not detected. As with CCL2 and CSF2, differences in MMP1 and MMP10 protein levels between CF and CTRL cells were not significant 8 h postinfection; nevertheless, in the CF cells these levels were higher and increased over time. In an *in vivo* model, MMP10 seemed essential in activating protective host responses initiated by the epithelium after *P*. *aeruginosa* infection [32]. However, it has been demonstrated that MMP10 shares with the neutrophil serine proteases an ability to activate MMP1 [33]. Although nothing is known about the role for MMP1 or MMP10 in CF, our data suggest that these two enzymes may be involved in tissue damage related to excessive inflammation and may contribute to infection-related CF pathology. We also detected significant differences in IL-8 and IL-6 production after infection in CF and CTRL cells. In lung samples from patients with CF, IL-8 and IL-6 levels were elevated and correlated with airway obstruction [34–37].

The increases in TLR2 and TLR4 expression may contribute to the proinflammatory response of CF cells. In one study, LPS recognition was sufficient to activate TLR2- and TLR4-dependent signaling in epithelial cells [38]. Similarly, increased TLR9 mRNA expression in infected CF cells may be clinically relevant, as TLR9 has been found to play a detrimental role in the pulmonary response to *P*. *aeruginosa* [39]. However, investigations of TLR protein expression would be of interest, as one study showed TLR6 mRNA expression without TLR6 protein expression in epithelial cell lines [40].

In contrast to the upregulated genes encoding cytokines and MMPs, the genes that were downregulated in CF cells in our study have not been previously identified as relevant in CF. Nevertheless, biological considerations suggest that downregulation of these genes may explain phenotypic features of CF. Adrenomedullin 2 (ADM2) protects against tissue injury in various organs including the lung, central nervous system, cardiovascular system, and kidney. In a rat model of testis inflammation, ADM2 treatment protected against LPS-induced damage by decreasing the levels of reactive oxygen species, TNF-α, IL-6, and IL-1 ß [41]. ADM2 attenuated the severity of ventilator-induced lung injury in mice [42] and, when used in low doses, protected mouse lungs from early ischemia/reperfusion injury by preserving the integrity of the blood-air barrier and by strongly decreasing the influx of leukocytes into the alveolar spaces [43]. ELF5 (also called ESE-2), an epithelial-specific member of the ETS transcription factor family, is expressed at high levels in the salivary glands, mammary glands, and trachea and at far lower levels in the lung [44]. ELF5 regulates the epithelium-specific gene K18 and may contribute to epithelial regeneration [45].

Other groups have performed studies to evaluate changes in the transcriptional response of CF compared to CTRL epithelial cells after *P*. *aeruginosa* infection. Differences in the experimental protocols preclude detailed comparisons of the results. However, the findings fall into two main categories. Thus, in one study, infected CF cells (IB3 cell line) overexpressed proinflammatory mediators such as cytokines, chemokines, and growth factors but underexpressed protease inhibitors, compared to CTRL cells (S9 cell line) [46]. Although the genes described do not exactly match those identified in our study, they belong to the same biological families. In contrast, another study produced contradictory results to ours, with higher levels of IL-8, IL-6, and ICAM-1 expression in CTRL cells (CFT1-LCFSN) compared to CF cells (CFT1-ΔF508) after infection [47].

The limitation in this study is the overall difference between the mean ages of the patients and CTRL, which may influences their response against *P*. *aeruginosa*. Influence of these parameters was not tested in our analysis since mutation status is shared with age (aged CTRL vs. young CF). Also, we choose to grow hAECBs in 2D monolayers for two main reasons. Firstly, CF cells grown at air-liquid interface produce more mucus than control cells that may prevent bacteria to physically interact with the cells and thus the subsequent cell host response. Also, introducing *Pseudomonas aeruginosa*, at the apical side of the cells, even in a reduce liquid volume would have resulted in the lost of the air-liquid interface.

In summary, we performed a comparative transcriptomic analysis to obtain a complete picture of the genes differentially regulated in CF versus CTRL respiratory epithelial cells in response to alive *P*. *aeruginosa* infection. Our results show that the CF respiratory epithelium responds to the presence of *P*. *aeruginosa* by producing, depending on the time of infection, an array of proteins that magnify the inflammatory response: thus, our protein analysis revealed that the infection induced overproduction of TNF, CSF2, CSF3, CCL2, MMP1, and MMP10 in CF cells compared to CTRL cells. Most of these proteins were produced by CTRL cells, but in lower levels. In contrast, after *P*. *aeruginosa* infection, some genes were activated in CTRL epithelial cells but not in CF cells. Interestingly, most of these genes are beneficial for tissue integrity. Our data on downregulated genes in infected CF cells may help to understand the mechanisms that underlie the persistence of *P*. *aeruginosa* in the CF lung. Furthermore, our identification of upregulated genes in infected CF cells may shed light on the mechanisms of the inflammatory response. We will now investigate the consequences of manipulating the expression of the genes that are differentially regulated in CF and CTRL cells, using markers for inflammation and antimicrobial activity as the endpoints. The expected results may suggest strategies for combatting *P*. *aeruginosa* infection, thereby improving both survival and quality of life in patients with CF.

## Supporting Information

S1 Table Characteristics of hAECBs. (XLSX)

S2 Table Lists of genes upregulated or downregulated in CTRL cells upon *P*. *aeruginosa* infection. We defined significant regulation as an at least 2-fold increase (tab: up-regulated genes CTRL cells) or decrease (tab: down-regulated genes CTRL cells) in expression after infection versus baseline (0 h), with *P*<0.001. ID, unique gene identifier; 0 h, nonstimulated; 4 h and 6 h, 4 and 6 hours postinfection; FC, fold change; adjpBH, adjusted *P* value to which the α cutoff was applied. **Lists of the 171 selected upregulated or 236 selected downregulated genes differentially expressed between CF and CTRL cells in response to *P*. *aeruginosa* infection:** For genes differentially expressed between CF and CTRL cells at baseline (0 h) and after infection, we computed the ratio of the FC value at each postinfection time point over the FC value at 0 h and then selected ratio ≥1.5 (upregulation: tab: upreg CF vs CTRL) and ≤0.6 (downregulation; tab: down reg CF vs CTRL), at one or more postinfection time points. For genes differentially expressed at a single time point or at two or three consecutive time points (2 and 4 h; or 2, 4 and 6 h; or 4 and 6 h), we selected FC≥2 (upregulation; tab: upreg CF vs CTRL) and FC ≤0.5 (downregulation; tab: down reg CF vs CTRL).
(XLSX)

S1 Fig Transcript abundance of innate immune receptors in CF and CTRL cells upon *P*. *aeruginosa* infection kinetic profiled by RNA-seq data. Normalized read count of Toll-Like Receptor (TLR), CD14 and Nod Receptors in CF and CTRL cells upon *P*. *aeruginosa*. Asterisks indicate statistically significant differences (*p<0.05; **p<0.01; § = p<0.001, #p<10e-8).
(TIF)
