# Supplementary figures and images for: Normal and Cystic Fibrosis Human Bronchial Epithelial Cells Infected with Pseudomonas aeruginosa Exhibit Distinct Gene Activation Patterns
Source: PLoS One. 2015 Oct 20;10(10):e0140979. doi: 10.1371/journal.pone.0140979 (PMC4618526; doi:10.1371/journal.pone.0140979)

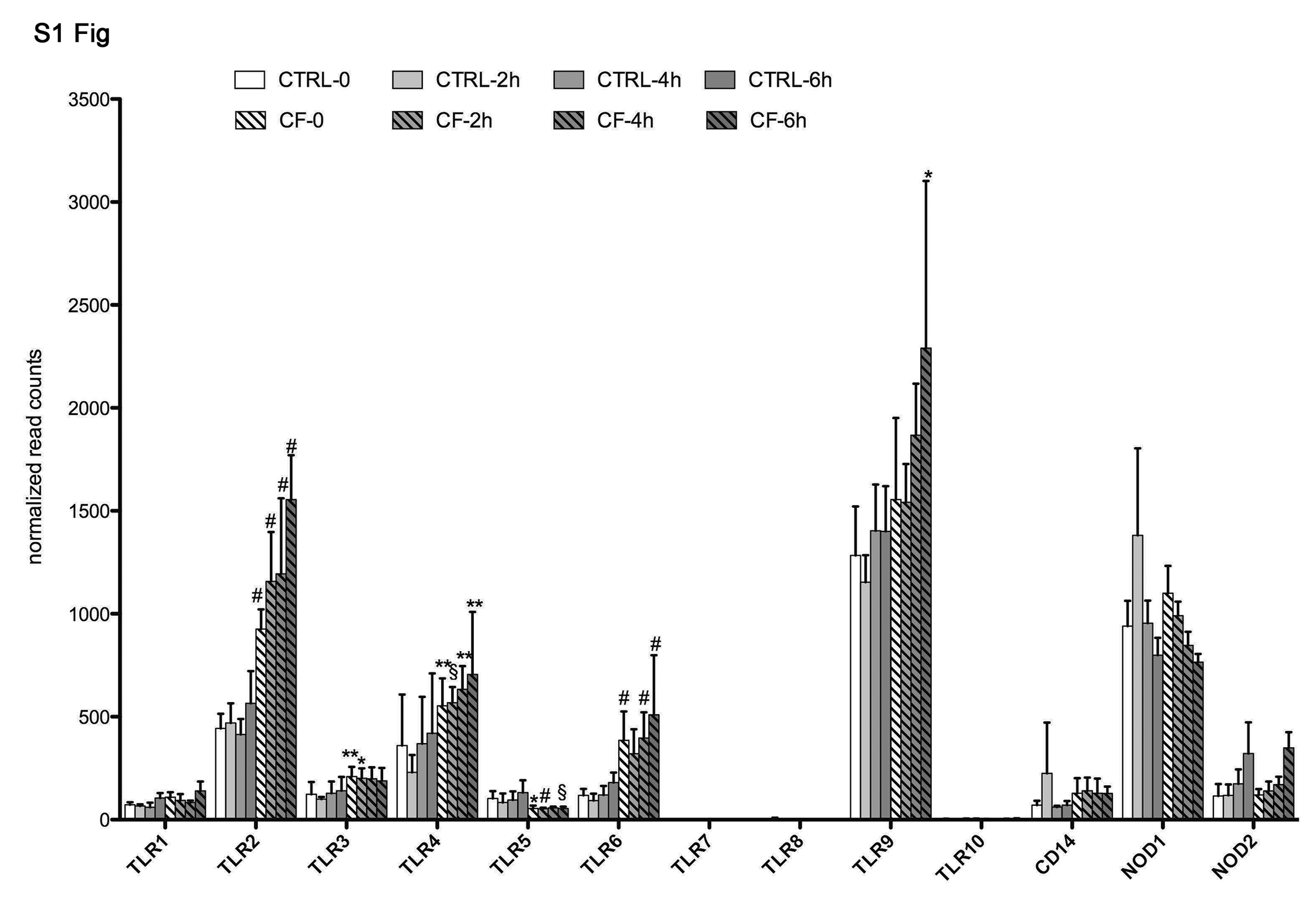

Supplement: S1 Fig — Normalized read count of Toll-Like Receptor (TLR), CD14 and Nod Receptors in CF and CTRL cells upon P. aeruginosa. Asterisks indicate statistically significant differences (*p<0.05; **p<0.01; § = p<0.001, #p<10e-8). (TIF) [file pone.0140979.s003.tif]
